# Pleomorphic adenoma of a subconjunctival ectopic lacrimal gland

**DOI:** 10.4103/0301-4738.77018

**Published:** 2011

**Authors:** Ravindra Kumar Chowdhury

**Affiliations:** Department of Ophthalmology, VSS Medical College Burla, Sambalpur, Orissa, India

Dear Editor,

The case report by Patyal *et al*.[1] on pleomorphic adenoma of a subconjunctival ectopic lacrimal gland was interesting and the authors deserve congratulations for reporting it. However, I wish to make the following observations on this rare case report:

1. Fig. 1 of the report points to the lesion protruding from the lateral canthus of the right eye, in contrast to the description part, where the authors have mentioned the right eye examination to be within the normal limit. This puzzled me and definitely it could have puzzled other readers also.
2. In the first paragraph of the report, they have mentioned that the growth is of the left eye, but the photographs clearly depict that the growth is coming out of the right eye.
3. In the discussion part, the authors could have added few lines about the frequency of the recurrence of pleomorphic adenoma of ectopic lacrimal gland at other sites.
